# Actinide and Lanthanide Adsorption onto Hierarchically Porous Carbons Beads: A High Surface Affinity for Pu

**DOI:** 10.3390/nano9101464

**Published:** 2019-10-16

**Authors:** Vittorio Luca, Devlet G. Sizgek, Erden Sizgek, Guilhem Arrachart, Cyrielle Rey, Nicholas Scales, Zaynab Aly, Glenna L. Drisko

**Affiliations:** 1 Comisión Nacional de Energía Atómica, Centro Atómico Constituyentes, Avenida General Paz 1499, San Martin 1650, Argentina; 2 A CSIRO Materials Science and Engineering, Bradfield Rd., Lindfield, NSW 2070, Australia; 3 ICSM, CEA, CNRS, ENSCM, Univ. Montpellier, 30207 Marcoule, France; guilhem.arrachart@cea.fr (G.A.); cyrielle.rey@cea.fr (C.R.); 4 Australian Nuclear Science and Technology Organization, New Illawara Road, Lucas Heights, NSW 2234, Australia; nsz@ansto.gov.au (N.S.); zax@ansto.gov.au (Z.A.); 5 CNRS, Univ. Bordeaux, Institut de Chimie de la Matière Condensée de Bordeaux, UMR 5026, 87 av du Dr. Schweitzer, 33600 Pessac, France; glenna.drisko@icmcb.cnrs.fr

**Keywords:** actinide and lanthanide sorption, carbon, hierarchical pore structures, polyacrylonitrile, phenolic resin

## Abstract

Structured carbon adsorbents were prepared by carbonizing macroporous polyacrylonitrile beads whose pores were lined with a mesoporous phenolic resin. After activation, the beads were tested for minor actinide (Np and Am), major actinide (Pu and U) and lanthanide (Gd) adsorption in varying acidic media. The activation of the carbon with ammonium persulfate increased the surface adsorption of the actinides, while decreasing lanthanide adsorption. These beads had a pH region where Pu could be selectively extracted. Pu is one of the longest lived, abundant and most radiotoxic components of spent nuclear fuel and thus, there is an urgent need to increase its security of storage. As carbon has a low neutron absorption cross-section, these beads present an affordable, efficient and safe means for Pu separation from nuclear waste.

## 1. Introduction

Carbon-based adsorbents are probably among the earliest materials known to have been deliberately used for purification of liquids [1]. Documented evidence suggests that more than 1500 years BC, the ancient Egyptians used forms of activated carbon for removing odours from festering wounds and from the intestinal track as well as for water purification [2]. Subsequent uses of carbons have included these and many other applications [3] and activated carbons have become the most ubiquitous materials for use in water purification worldwide, even though they are costly [4]. A particularly important application is the adsorption of radionuclides for environmental remediation [5,6], nuclear waste management [7,8,9,10], and biomedical applications [11]. In activated carbons, van der Waals forces are mainly responsible for surface adsorption, thus, carbon nanotubes, which have demonstrated chemisorption resulting in high adsorption capacities, are starting to attract interest [8]. However, such materials cost hundreds of dollars per gram [8], and therefore, the availability of a selective yet inexpensive adsorbent would be advantageous.

Activated carbons are generally prepared through the slow heating of a carbon source up to 600 °C in an inert environment, followed by either chemical activation (with an oxidant) or physical activation at high temperatures [1]. The resulting materials are normally composed of disordered micropores. In order to allow access to larger molecular species, it is necessary to be able to tailor the pore dimensions of the substrate [12]. First attempts to produce porous carbons using templates began in 1986 using mesoporous silica [13]. Then, in 1992, zeolites were infiltrated with acrylonitrile, followed by polymerization, carbonization and finally, template dissolution [14]. Since these initial reports, nanocasting of other hard templates has been thoroughly explored, generating a range of microporous and mesoporous materials [15,16,17,18,19,20]. The main drawback of this approach is the difficulty involved in removing the hard template, requiring the use of strong acids or bases that can affect the activity of the carbon material.

Soft templating using polymers and surfactants to produce porosity is another approach. Indeed, this was performed using polymer blends as long ago as 1997 by Ozaki et al. who used blends of carbonizing and decomposing polymers to prepare materials with micro- and mesoporosity [21]. Other procedures have subsequently been developed in which combinations of block copolymer templates and phenolic or resorcinol formaldehyde resin precursors are used to generate the carbon structure [22,23]. The self-assembly and supramolecular interactions between the amphiphilic block copolymers and the organic resin are analogous to the inorganic building blocks in metal oxide self-assembly templating. All these carbon-based porous materials have become popular materials for study [24]. Whether any of the carbon mesophases that have been generated in the past decade have any advantageous properties that distinguish them in application terms from commercially available activated carbons or mesophase pitches, such as Mitsubishi’s ARA24, is not known. However, it is anticipated that larger pores would allow for the accommodation and performance of larger molecules, such as proteins or enzymes.

Many carbon spheres have been previously reported [25], but the materials reported here are unique in both bead size and pore structure. Polyacrylonitrile (PAN) has been used as a structure-directing agent to produce macroporous oxide-PAN composites [26,27,28,29,30]. The process for the preparation of the hierarchical beads is very relevant to industrial applications, since PAN is an inexpensive material that accounted for 4–6% of total commercial fiber production in 2000 [31]. We have previously prepared zirconium titanate beads with hierarchical porosity that are suitable for aqueous phase metal extraction by combining PAN, which can easily be formed into beads having novel radial macroporosity, with block copolymers that generate mesoporosity [32]. We have shown that such materials display enhanced mass transport properties while at the same time retaining the high surface areas and uniform pores in the micro- and mesoporous range [32,33]. Both crystallization and surface capping with bisphosphonates produce highly acid-resistant materials [34]. We have also shown that pores at the higher end of the mesopore regime give enhanced diffusion kinetics [33,35].

Our primary interest in this communication is the synthesis of carbon materials with hierarchical porosity in the form of millimetre-sized beads suitable for large-scale applications in the separation of radioactive and non-radioactive heavy metal species. We studied the surface affinity of the radioactive species towards the carbon. Although porous activated carbons are well known adsorbents, the selectivity they possess for the adsorption of actinides has, to the best of our knowledge, never been closely examined. Here, we compare the acid dependence for the adsorption of tracer levels of lanthanides and actinides. The separation of lanthanides from actinides, of major actinides from minor actinides and of uranium and plutonium is currently very difficult. We show that carbon beads derived from the pyrolysis of PAN beads maintain their structure and that the surfaces can be subsequently coated with a carbon film with large mesopores (10 nm). These carbon bead adsorbents have the ability to efficiently separate plutonium from other actinides and lanthanides under given conditions.

## 2. Materials and Methods

### 2.1. Materials

The following chemicals were purchased from Sigma Aldrich (Castle Hill, NSW, Australia): Brij 58 (B58), 150,000 molecular weight poly(acrylonitrile), hydrochloric acid, sodium hydroxide, phenol, formaldehyde, ammonium persulfate (NH_4_)_2_S_2_O_8_ (APS) and anhydrous dimethyl sulfoxide. Uranyl nitrate hexahydrate was obtained from Merck (Bayswater, VIC, Australia). ^153^Gd (>99% purity) was obtained from Perkin-Elmer Life Sciences (Rowville, VIC, Australia) as a 37.0 MBq (=1 mCi) solution in 2 mL total volume. The Np and Pu stocks were in the form of nitrate salt solutions prepared in-house. This was carried out by heating metals or metal oxide powders in nitric acid. A small amount of fluoride was added to help the dissolution. Silicon oil (Fluka) was used as received.

### 2.2. Bead Synthesis

The present hierarchical carbon beads were prepared in two steps with the application of a pyrolysis procedure after each step. A 6 wt% poly(acrylonitrile) (PAN) solution was prepared by dissolving 3 g of polymer powder in 47 g of dimethyl sulfoxide (DMSO) at 40 °C overnight. Brij 58 (0.82 g) and silicon oil (0.5 mL) were added to this solution, which was sonicated and then incubated at 32 °C. The solution was passed through an automatic droplet generator using needles of 21 gauge. The free-falling droplets solidified on contact with a bath. The bath was prepared by dissolving 1 g of Triton-X100 surfactant in 5 L of deionized water. The precipitated PAN beads were washed with deionized water until all DMSO was removed and subsequently dried at 35 °C under 60–90% relative humidity for 2 days. The beads were stabilized by heating in air at a rate of 1 °C/min to 230 °C and maintained at this temperature for 3 h. The system was cooled and purged with nitrogen for a minimum of 2 h and then re-heated under an inert argon atmosphere at a rate of 1 °C/min to 700 °C and maintained at this temperature for 3 h. These beads were used as the macroporous structure upon which the mesoporous layer was deposited in the second step.

Low molecular weight ethanol-soluble phenolic resin (resol) was prepared from phenol and formaldehyde using a base-catalyst via the methodology reported by Meng et al. [23]. In this procedure, 5 g of phenol was dissolved in 1.06 g of 20 wt% sodium hydroxide at 40–42 °C. After 10 min, 8.6 g of 37% formaldehyde was added dropwise, ensuring that the temperature of the reaction mixture remained below 50 °C. After 1 h at 70–75 °C, the reaction mixture was cooled to room temperature and the pH adjusted to pH 7.0 using 0.6 M HCl. The final molar ratio of phenol:formaldehyde:NaOH was 1:2:0.1. The PAN beads were placed in a tube that was evacuated and then the previously prepared F127-B58 resol was added to the beads and allowed to infiltrate the pores. After soaking overnight, excess resol was removed by filtration and the beads were dried and then subjected to the same thermal treatment that was applied to the PAN beads.

When the Pluronic F127 was used as the sole template following a literature report [23], a disordered mesophases was consistently produced. In order to produce an ordered mesophase, a co-templating approach was found to be necessary, in which the F127 was spiked with a small amount of Brij 58 at an F127:B58 molar ratio of 1:0.022 by dissolving the F127 and B58 in 100 g of ethanol and adding this to the phenolic resin solution prepared in the manner described above until a homogeneous solution was obtained. The final solution was evaporated under vacuum at 50 °C until a gel was obtained. A portion of the gel was coated on a glass slide and variously examined by small angle X-ray diffraction over a 40-day period as a function of curing temperature.

Surface activation of hierarchical carbon beads to give activated hierarchical carbons beads was achieved through an oxidation reaction. In a typical reaction, 500 mg of hierarchical carbon beads was added to 30 g of APS in H_2_SO_4_ (2 mol/L). The mixture was stirred at 60 °C for 24 h and subsequently filtered. The beads were washed several times with water until sulfates could no longer be detected in the wash water, and dried overnight in a vacuum oven at 80 °C.

### 2.3. Characterization

Small angle X-ray scattering (SAXS) measurements were performed on a Bruker Nanostar SAXS camera (Preston VIC Australia), with pin-hole collimation for point focus geometry. The instrument source was a copper rotating anode (0.1 mm filament) operating at 50 kV and 24 mA, fitted with cross-coupled Göbel mirrors, resulting in Cu Kα radiation of wavelength 1.54 Å. The SAXS camera was fitted with a Hi-star 2D detector (effective pixel size 100 µm). The sample to detector distance was chosen to be 300 mm, which provided a q-range of 0.03 to 0.69 Å^-1^ (q = (4πsinθ)/λ where θ is the scattering angle and λ is the wavelength of the incident X-rays).

Nitrogen adsorption/desorption isotherms were measured at 77 K on a Micromeritics ASAP 2010 unit (Gosford, NSW, Australia). All the samples were out gassed at 150 °C under vacuum for 24 h prior to measurement. The surface area was calculated using the Brunauer–Emmett–Teller (BET) method. For pore size distribution calculations, the Barrett–Joyner–Halenda (BJH) method using the adsorption branch was used, which is part of the DFT Plus software of ASAP 2010.

Fourier transform infrared (FTIR) spectra of the phases were recorded in KBr (10% w/w) in the range 4000–650 cm^−1^ with a Nicolet Nexus 8700 FTIR spectrometer (Goodwood, SA, Australia).

A JEOL 6400 scanning electron microscope (SEM) (Frenchs Forest, NSW Australia) was used to investigate the bead morphologies. For the SEM investigation, individual beads were sectioned with a razor to reveal the internal structure and were mounted on aluminum stubs using carbon adhesive tape before being coated with a plasma of gold.

Transmission electron microscopy (TEM) was conducted using a JEOL 2000FXII (Frenchs Forest, NSW Australia) instrument operating at 200 keV. TEM specimens were prepared by lightly grinding a small amount of powder in ethanol to form a suspension that was dropped by pipette onto holey-carbon-coated copper TEM grids that were allowed to dry in air.

Radioisotope adsorption: The adsorption experiments involved placing the beads in contact with a nitric acid media (0.0001–6 mol/L) spiked with tracer-level radionuclides. The beads (0.03 g contained in 7 mL polypropylene vials) were in 3 mL of solution for a volume-to-mass ratio of 100 mL/g for 2 h at room temperature, with thorough mixing via gentle mechanical agitation. Three separate adsorption experiments were carried out, grouped according to analytes ^153^Gd (160 cps), ^241^Am (60 cps), ^238^U (1 ppb), ^237^Np (2.4 ppb) and ^239^Pu (2.2 ppb). Post-contact, stocks and supernatants were filtered through hydrophilic 0.45 μm syringe filters (Sartorius) (Dandenong South, VIC Australia). The analytes were quantified with either a Perkin Elmer Wizard^2^ 2480 gamma counter (^153^Gd and ^241^Am) (Rowville, VIC, Australia) or a Perkin Elmer-SCIEX *Elan* 6000 Inductively Coupled Plasma Mass Spectrometer (ICP-MS) (^238^U, ^237^Np and ^239^Pu) (Rowville, VIC, Australia).

Calibration for ^238^U ICP-MS measurements was achieved with NIST-certified ICP-MS standards and its accuracy verified with independently certified standards. ^237^Np and ^239^Pu stock concentrations were quantified with an ORTEC^®^ Octet alpha spectrometer (Canberra, ACT Australia) from diluted stock aliquots evaporated onto stainless steel planchets. The alpha energies and counting efficiency were calibrated with a NIST-certified electrodeposited alpha spectrometry standard (Eckert & Ziegler Analytics, Atlanta, GA).

The quantity of radioisotope adsorbed was calculated in accordance with a previous report [36]. The adsorption calculation is based on a difference in concentrations between the initial solution and the concentration after a specified contact time, as given by Equation (1):

(1) $$qt=\frac{V\left(Ci-Cf\right)}{m}$$

where *q_t_* is the quantity of adsorbed radionuclide (mmol/g) at time *t* = 2 h, *V* is the volume of solution (L), *Ci* (mmol/L) is the concentration of vanadium ions in solution before contact, *Cf* (mmol/L) is the concentration after *t* minutes of contact, and *m* is the mass of the adsorbent material (g) in solution.

## 3. Results

### 3.1. Preparation and Characterization

**Figure 1:**
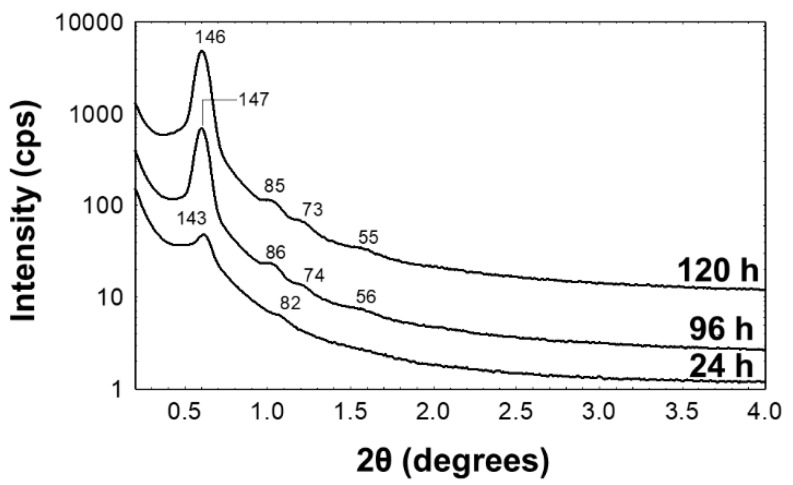
SAXD patterns of the F127-Brij 58 phenolic resin thick films cured for different times (**a**) 24, (**b**) 96 and (**c**) 120 h.

In order to achieve the preparation of hierarchical porous carbon beads with large mesopores, we selected the triblock copolymer F127 as the primary porogen. The use of F127 with phenol:formaldehyde:NaOH molar ratios of 1:2:0.1, as previously reported [23], consistently yielded poorly ordered materials in our laboratory, that showed no reflections in the low-angle X-ray diffraction pattern and no evidence of ordered mesophases in the transmission electron microscope images. However, it was possible to prepare ordered films using a dual template approach in which F127 was spiked with Brij 58. Synergistic mixtures of surfactants result in lower critical micellization concentrations and changes to interfacial tension [37]. Brij 58 has a long hydrocarbon chain that lowers the surface area of its interaction with water. The increased polarity between the block copolymer/surfactant mixture was likely responsible for the better order within the structure. SAXD patterns of thick phenolic resin films cured at 100 °C for increasing time periods are shown in Figure 1. The strong primary reflection at a d-spacing of 150 Å and the four weak higher angle reflections, indicate the formation of a well ordered mesophase. It is apparent from the increased intensity of the higher order reflections that the degree of mesophase ordering improved as the curing time increased. The sample cured for 120 h (Figure 1c) had d-spacings at 146, 85, 73 and 55 Å that correspond well with a hexagonal unit cell with a value of 169 Å. The original synthesis by Meng, et al. found a lattice parameter a of 139 Å [23].

**Figure 2:**
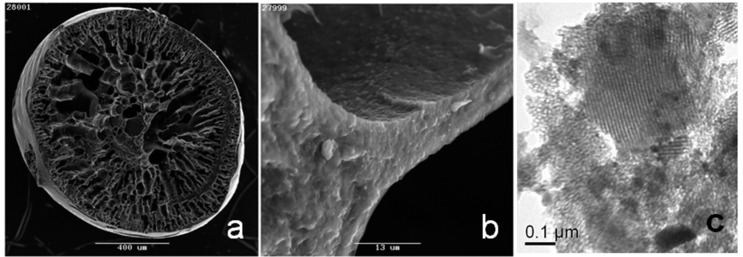
SEM images of hierarchical carbon beads, at (**a**) low and (**b**) high magnification, and (**c**) TEM image of the hierarchical carbon beads.

The SEM images of the hierarchical composite carbon beads prepared by infiltrating the PAN-derived carbon beads with F127-B58 phenolic resin are shown at low and high magnification in Figure 2a,b. The beads after final pyrolysis in argon at 700 °C had a smooth outer surface. Internally, they displayed the characteristic radial macroporosity of PAN. At high magnification, the surfaces of these internal macropores appeared to be comprised of sub-micron particles. Detailed TEM examination of the composite beads showed the presence of both ordered and disordered mesopores (Figure 2c). Much of the specimen consisted of rather amorphous material, originating from the macroporous PAN walls. However, fringes that were characteristic of ordered mesophase material deriving from incorporated resol were relatively easily located.

**Figure 3:**
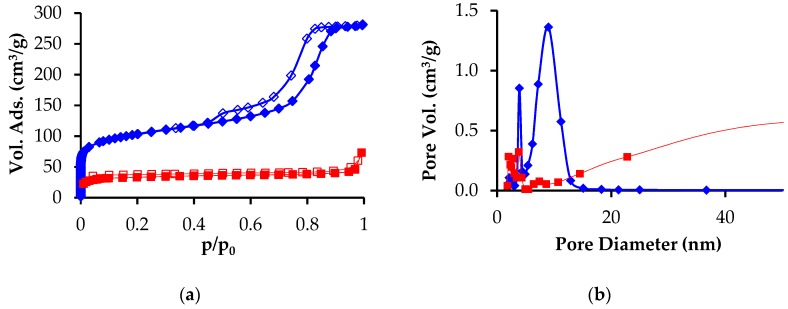
(**a**) Nitrogen adsorption-desorption isotherm and (**b**) pore size distribution of the PAN beads before (red squares) and after (blue diamonds) resol infiltration, measured at 77 K.

The nitrogen adsorption-desorption isotherm and pore size distribution of the carbonized PAN and PAN-phenolic resin beads are shown in Figure 3. The composite displays a typical Type IV capillary condensations step at p/p_0_ ~0.8, indicative of large mesopores, and also significant microporosity at p/p_0_ < 0.1. The pore size distribution clearly shows the existence of bimodal mesopores with dimensions of about 6 and 10 nm. PAN beads prepared without incorporating mesoporosity via resol infiltration had a much smaller mesopore volume and diameter (Figure 3b). Specifically, the PAN beads had a cumulative pore volume (between 1.7 and 300 nm) of 0.06 cm^3^/g, where the resol-PAN beads displayed 0.39 cm^3^/g. The PAN bead surface area was only 98 m^2^/g, whereas the hierarchical carbon beads had a comparatively large surface area of 341 m^2^/g.

The data presented in Figure 1, Figure 2 and Figure 3 clearly demonstrate that infiltration of the PAN-derived carbon beads with F127-B58 containing resol, followed by pyrolysis, resulted in a coating of mesoporous carbon on the macropore surfaces of the initial PAN-derived beads. The approach used here is advantageous for many reasons. The PAN bead platform allows for the preparation of macroscopic bead materials with radial macroporosity that facilitate mass transport and deployment in fixed bed column applications. The incorporation of mesoporous carbon material on the walls of the macroporous beads generates a dramatic increase of surface area and makes it possible to tailor pore dimensions and modify the pore chemistry through functionalization. Naturally, in order to use this high surface area to the maximum extent, it is first usually necessary to activate the carbon surfaces.

**Figure 4:**
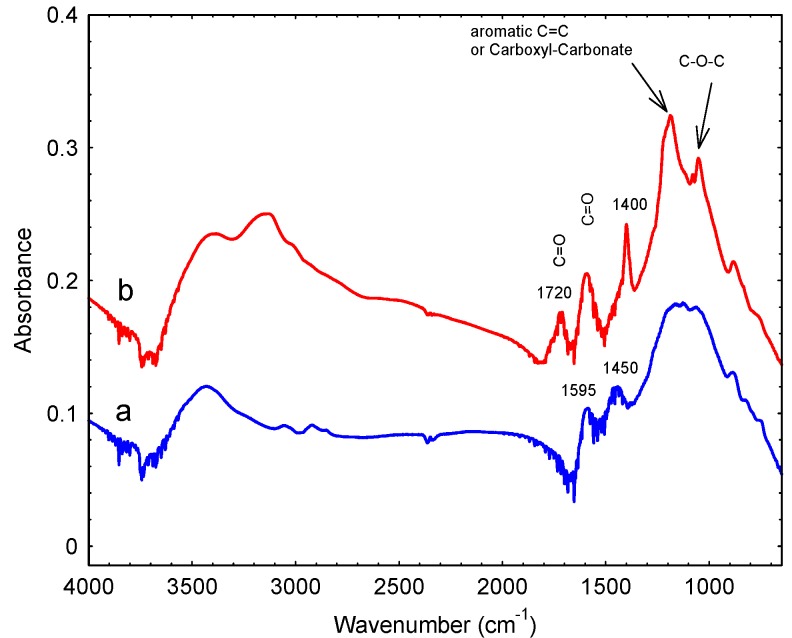
FTIR spectra of (**a**) hierarchical carbon beads and (**b**) activated hierarchical carbons beads after activation with APS.

Many methods are available for activating the surfaces of carbon materials. In the present study, APS was used as per previously described procedures [38]. The FTIR spectra of as-prepared hierarchical carbon beads are compared with that of APS-treated beads in Figure 4. On APS treatment, several new bands were generated relative to the untreated samples. Most notable are the additional bands at 1053, 1186, 1400, 1720 and 3147 cm^−1^. These bands can be assigned as follows in Table 1, based on [39].

The FTIR data makes it apparent that treatment with APS generated surface carboxyl groups. This was confirmed with the observation of a band at around 1720 cm^−1^, which can be assigned to C=O stretching vibrations of carboxyl groups, and the broad band appearing at around 3150 cm^−1^ which can be also attributed to the formation of carboxylic structures [40].

### 3.2. Adsorption of Lanthanides and Actinides

A preliminary assessment of the ability of the hierarchical carbon bead materials to adsorb actinides was undertaken by placing the beads in contact with an acidic solution of a given radioisotope tracer. The very low concentration of the target isotope relative to the concentration of H^+^ allowed for an evaluation of the selectivity of the carbon surfaces. Tracer quantities were used to avoid the occurrence of precipitation upon large changes in pH. Thus, adsorption enhancement should be due to true surface affinity by the cations.

**Figure 5:**
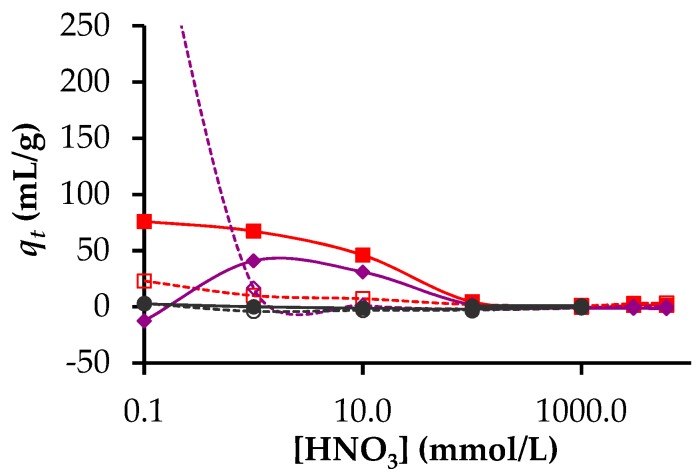
Distribution coefficients for neptunium, gadolinium and americium adsorption as a function of acid concentration. ^237^Np adsorption on hierarchical carbon beads (**○**) and activated hierarchical carbons beads (●), ^153^Gd adsorption on hierarchical carbons beads (◇) and activated hierarchical carbons beads (◆), ^241^Am adsorption on hierarchical carbons beads (☐) and activated hierarchical carbons beads (■).

Figure 5 shows that neither hierarchical carbon beads nor activated hierarchical carbons beads possessed any significant affinity for ^243^Np^3+^. In contrast, both carbon materials showed some capacity for the adsorption of both ^153^Gd^3+^ and ^241^Am^3+^ at acid concentrations less than 0.01 mol/L (Figure 5). For the beads that had not been activated, a *q_t_* value of 400 mL/g was obtained for ^153^Gd^3+^ adsorption from the solution with an acid concentration of 0.1 mmol/L. This decreased to values close to zero for acid concentrations greater than 1 mmol/L acid. For the activated beads, low *q_t_* values were obtained for ^153^Gd^3+^ adsorption in the concentration range of 100 to 1 mmol/L. At 0.1 mmol/L acid, no ^153^Gd^3+^ adsorption was observed for the activated beads.

In the case of ^241^Am^3+^, appreciable adsorption was observed only for the activated beads and only for acid concentrations < 100 mmol/L reaching a maximum at 0.1 mmol/L. The dependence of adsorption on solution pH for the present activated beads is similar to that recently reported by Wang et al. [41] for Am^3+^ adsorption on multiwall carbon nanotubes (MWCNT). In contrast to the use of MWCNT, the present macroscopic bead materials make Am^3+^ separation possible using much cheaper materials without the need to undertake the relatively complex ultrafiltration operation. We also show here that at an acid concentration of 0.1 mmol/L (pH = 4) or lower, it would appear feasible to separate a minor actinide from a lanthanide, although this has been observed previously [42].

**Figure 6:**
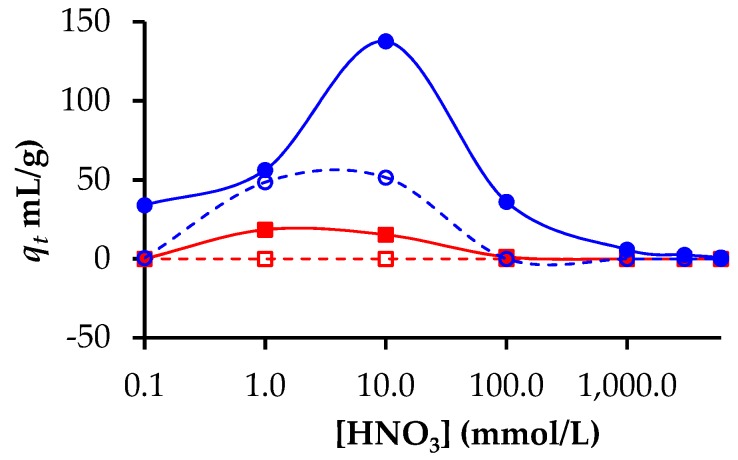
Distribution coefficients as a function of acidity for Pu adsorption on hierarchical carbon beads (○) and activated hierarchical carbons beads (●), U adsorption on hierarchical carbon beads (☐) and activated hierarchical carbons beads (■).

The adsorption of the major actinides uranium and plutonium as UO_2_^2+^/UO_2_OH^+^ and PuOH^3+^/(PuOH^3+^)_n_ was also studied. The acid dependence for Pu and U adsorption is shown in Figure 6. Both non-activated and activated beads showed an adsorption maxima at an acid concentration of 10 mmol/L, although the highest *q_t_* was obtained for the activated materials. These activated beads showed appreciable adsorption at acid concentrations as high as 100 mmol/L. At acid concentrations of 100 mmol/L, 27% of the plutonium was removed from the solution. In this range of acid concentration, the other elements were poorly extracted by the beads (see Table 2). On the assumption that all lanthanides behave similarly, the present initial results suggest that at an acid concentration between 10 and 100 mmol/L, the present materials could be used to selectively extract plutonium in the presence of both other actinides and lanthanides.

## 4. Conclusions

Carbon materials with hierarchical porosity have been prepared using polyacrylonitrile-templated carbon beads coated with block copolymer templated mesoporous phenolic resin to produce stable carbon materials with high surface areas and radial macroporosity that have been proven to facilitate rapid mass transport [33]. The surface chemistry could be tailored using APS, which dramatically changed the sorption properties of the beads.

The selectivity of these carbon materials for various lanthanides and minor and major actinides has been evaluated against [H^+^]. Americium is often taken as a representative of all the actinides [41,42]; however, we found very distinct behaviour between the actinides studied. The materials displayed negligible capacity for the adsorption of ^243^Np^3+^ in the acid concentration range of interest. However, for [H^+^] < 0.1 mol/L, modest distribution coefficients of about 50 mL/g were measured for ^153^Gd^3+^ and ^241^Am^3+^. With regards to separation of the major actinides U and Pu, it was found that at an acid concentration of 100 mmol/L, Pu could be adsorbed with *q_t_* values of 150 mL/g. Thus, our results show that Pu can be selectively removed in the presence of other lanthanides and uranium and minor actinides. Such separation technology is currently lacking but is greatly needed to increase the safety of waste treatment, whether we pursue reprocessing or secure burial.

In the future, radioisotope leaching from these beads will be examined. Additionally, the separation factors of the beads will be determined by measuring adsorption of actinides and lanthanides in a mixed solution.

## Figures and Tables

**Table 1:** Fourier transform infrared (FTIR) band assignments according to Fanning et al. [39].

Band Position (cm^−1^)	Assignment
1053	Alcohol groups
1186	Alcohol
1400	Lactones
1595	Quinones
1720	COOH
3147	COOH

**Table 2:** The % removal of radioisotopes as a function of nitric acid concentration by PAN-phenolic resin beads (hierarchical beads) and PAN beads after carbonization and activation.

Radioisotope	% Removal at 1 mmol HNO_3_		% Removal at 10 mmol HNO_3_		% Removal at 100 mmol HNO_3_	
	Hierarchical beads	PAN beads	Hierarchical beads	PAN beads	Hierarchical beads	PAN beads
^241^Am	40	9	31	7	4	2
^153^Gd	29	14	23	1	2	0
^237^Np	0	0	0	0	0	0
^238^U	13	10	11	9	1	9
^239^Pu	36	50	22	51	27	0
